# Serum 25-hydroxyvitamin D level is associated with short-term glycemic variability metrics derived from continuous glucose monitoring in T2DM

**DOI:** 10.1038/s41598-023-45846-1

**Published:** 2023-10-27

**Authors:** Guohong Zhao, Xinwen Yu, Lin Wang, Yuxin Jin, Aili Yang, Fei Sun, Xin Wang, Xiaorui Jing, Bin Gao

**Affiliations:** 1 Department of Endocrinology, Tangdu Hospital, Air Force Medical University, Xi’an, 710038 Shaanxi Province People’s Republic of China; 2 College of Medicine, Xi’an International University, Xi’an, 710077 Shaanxi Province People’s Republic of China; 3 Engineering Research Center of Personalized Anti-Aging Health Product Development and Transformation, Universities of Shaanxi Province, Xi’an, 710077 Shaanxi Province People’s Republic of China

**Keywords:** Endocrine system and metabolic diseases, Endocrine system and metabolic diseases

## Abstract

This study aims to investigate the association between 25-hydroxyvitamin D (25OHD) and continuous glucose monitoring-assessed short-term glycemic variability (GV) and HbA1c among patients with type 2 diabetes mellitus (T2DM). We conducted a cross-sectional study recruiting 325 patients. The association between 25OHD and GV metrics (mean amplitude of glycemic excursions [MAGE], coefficient of variation [CV], standard deviation of sensor glucose [SD], and TIR) and HbA1c were analyzed using multivariable linear and logistic regression analyses. The 25OHD level and GV metrics showed significant differences among HbA1c groups (*P* < 0.01). CV, MAGE, SD and HbA1c decreased, and TIR increased with ascending 25OHD tertiles (*P* < 0.05). Serum 25OHD was inversely associated with CV (β = − 0.211 [− 0.350 to − 0.071], *P* < 0.01) and HbA1c (β = − 0.061 [− 0.114 to − 0.031], *P* < 0.01), and further multivariable analyses confirmed these results (*P* < 0.05). However, no association of HbA1c and 25OHD was found with the highest tertile of CV. These findings revealed that increased GV and HbA1c were both associated with lower 25OHD, and the relationship between HbA1c and 25OHD was attenuated with higher glucose CV in T2DM. Taken together, the analyses suggest that increasing vitamin D status has effects on improvements in long-term glycemic control and low glycemic variability.

## Introduction

Type 2 diabetes mellitus (T2DM) is a major public health problem with an estimated global projection of 783 million cases by 2045. Evidences have demonstrated the importance of dietary vitamin components in the prevention and treatment of T2DM. Bioactive vitamins of these foods have been widely explored for their potential antioxidant, anti-apoptotic, and anti-inflammatory effects. Among them, vitamin D, which comes from a healthy diet and oral supplements, has become a hot topic for its potential prevention and treatment of diabetes and other chronic conditions^1–3^.

25-hydroxyvitamin D (25OHD), as a biomarker of serum vitamin D, plays an important role in T2DM. Higher serum 25OHD levels were significantly associated with lower all-cause and CVD mortality^4^. Longitudinal cohort studies proved an approximately 40% increased risk for incident diabetes in the lowest versus the highest category of 25OHD level^5^, suggesting a potential benefit of vitamin D in maintaining euglycemia. Vitamin D in maintaining glucose homeostasis has been proven in nondiabetic and prediabetic persons, but clinical studies in T2DM were inconclusive^6–8^. The indicators of glycemic homeostasis vary from study to study, even though fasting glucose, insulin, and HOMA-IR were significantly decreased after vitamin D intervention^9^. Several studies have proven that intervention duration, dose of vitamin D, ethnicity, BMI, baseline vitamin D and HbA1c are influencing factors for this inconsistency. To date, most studies have focused on the association between vitamin D and long-term glucose control^10–13^, which means that the effects of vitamin D on glycemic control are slow and sustainable. However, there is still a lack of in-depth research on the correlation between short-term blood glucose variability (GV) indicators and vitamin D. Notably, short-term glucose fluctuation in patients with diabetes can also generate long-term adverse effect^14,15^.

Continuous glucose monitoring (CGM) technology is dramatically expanding the dimensions of blood glucose, thereby complimenting HbA1c for diabetes management. Primary variables to characterize glycemic variability included the SD of glucose, mean amplitude of glycemic excursions (MAGE), coefficient of variation (CV) and time in range (TIR). There is increasing evidence on the association between CGM-assessed GV and the development of T2DM complications and an increased risk of cardiovascular events such as heart attacks and strokes^16^. Based on this, glycemic variability should be taken into account when exploring the correlation between vitamin D and glycemic control. Until now, there is still few studies focused on this field. Thus, in this study, we aimed to investigate the association between 25OHD and CGM-assessed GV among type 2 diabetes patients to provide clinical evidence of vitamin D for T2DM prevention and treatment.

## Results

### Characteristics of the participants

**Figure 1:**
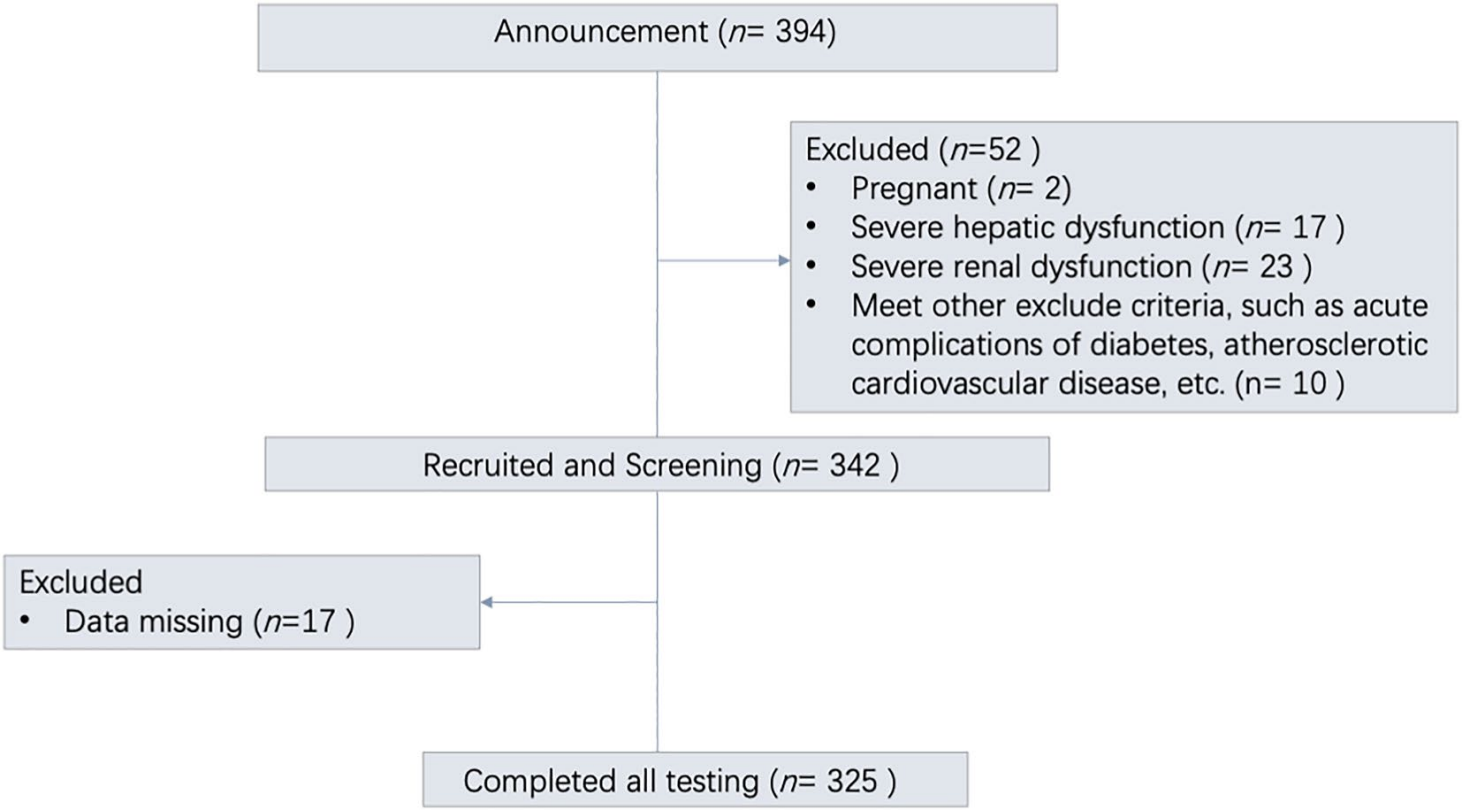
Flow diagram of participants included in the study.

A flow diagram of the participants is shown in Fig. 1. Table 1 presents the baseline characteristics of participants stratified by HbA1c. The study comprised 325 individuals with a mean age of 55.18 ± 13.20 years. A total of 68.8% of participants were male. The median diabetes duration was 6 years, and the mean HbA1c level was 8.74 ± 2.11%. The serum 25OHD concentration showed a significant downward trend with increasing HbA1c (*P* = 0.001). There were significant differences among different HbA1c levels in age, diabetes duration, TC, TG, mean sensor glucose, albumin, serum creatinine, eGFR and history of atherosclerotic cardiovascular disease. For history of drug usage, the metformin, RAAS inhibitors, calcium channel blockers and lipid-lowering agents were significantly different in HbA1c groups, but no difference in HbA1c levels was found between the basal insulin and basal/bolus insulin regimens. Along with HbA1c elevation, SD, MAGE, and CV level increased significantly, while TIR declined significantly (*P* < 0.001).

**Table 1:** Characteristics of study subjects by HbA1c.

Variables	All subjects (N = 325)	HbA1c < 7% (N = 73)	HbA1c:7–9% (N = 128)	HbA1c > 9% (N = 124)	*P* value
Age (years)^a^	55.18 ± 13.20	57.51 ± 11.52	57.43 ± 12.41	51.48 ± 14.14	< 0.001
Male sex^b^ (n)	225 (68.8%)	45 (61.6%)	93 (72.2%)	85 (68.5%)	0.270
Diabetes duration (years)^c^	6 (2.00–11.00)	6 (2.00–10.50)	8 (4.25–11.00)	5 (1.00–10.00)	0.001
SBP (mmHg)^a^	124.66 ± 10.43	124.01 ± 10.62	124.31 ± 9.13	125.40 ± 11.56	0.596
DBP (mmHg)^a^	73.48 ± 7.14	74.15 ± 7.60	73.58 ± 6.32	72.98 ± 7.66	0.527
BMI (kg/m^2^)^a^	25.07 ± 3.70	24.47 ± 4.12	25.50 ± 3.26	24.99 ± 3.85	0.157
TC (mmol/L)^a^	4.50 ± 1.08	4.27 ± 1.11	4.32 ± 0.95	4.81 ± 1.11	< 0.001
TG (mmol/L)^a^	1.75 ± 1.14	1.53 ± 0.94	1.68 ± 1.06	1.96 ± 1.30	0.026
ALT (U/L)^c^	29 (20.50–40.00)	27 (20.00–36.00)	30 (22.25–44.00)	28 (17.00–39.75)	0.071
AST (U/L)^c^	24 (19.00–32.50)	24 (20.00–29.00)	25 (20.00–33.75)	24 (18.00–33.00)	0.514
GGT (U/L)^c^	23 (17.00–34.00)	21 (16.00–32.00)	23 (17.00–35.75)	24.5 (17.00–34.00)	0.568
Albumin (g/L)^a^	44.39 ± 4.25	45.43 ± 3.57	44.55 ± 4.00	43.62 ± 4.72	0.013
Globulin (g/L)^a^	28.57 ± 4.13	28.82 ± 3.71	28.62 ± 4.32	28.38 ± 4.19	0.755
BUN (mg/dL)^a^	5.37 ± 1.65	5.23 ± 1.37	5.49 ± 1.85	5.32 ± 1.60	0.518
SCr (μmol/L)^a^	66.76 ± 18.45	69.51 ± 16.74	69.62 ± 20.89	62.20 ± 15.76	0.002
Uric acid (μmol/L)^a^	320.75 ± 89.95	333.45 ± 92.05	319.34 ± 88.44	314.73 ± 90.23	0.361
eGFR (mL/min/1.73 m^2^)^a^	133.49 ± 35.80	123.99 ± 27.34	127.79 ± 30.13	144.97 ± 42.20	< 0.001
Metrics of CGM					
MAGE (mmol/L)^a^	4.19 ± 2.17	3.18 ± 1.42	4.14 ± 2.07	4.83 ± 2.40	< 0.001
CV (%)^a^	20.76 ± 6.82	18.69 ± 5.51	20.05 ± 6.87	22.70 ± 7.01	< 0.001
SD (mmol/L)^a^	1.81 ± 0.81	1.36 ± 0.47	1.76 ± 0.76	2.12 ± 0.88	< 0.001
TIR (%)^a^	84.82 ± 17.50	95.78 ± 6.23	85.87 ± 15.71	77.28 ± 20.00	< 0.001
Mean sensor glucose levels (mmol/L)^a^	8.56 ± 1.65	7.26 ± 1.09	8.67 ± 1.44	9.21 ± 1.71	< 0.001
25OHD (ng/mL)^a^	13.18 ± 5.29	14.77 ± 6.03	13.55 ± 5.74	11.87 ± 3.89	0.001
ASCVD, n (%)^b^	67 (20.6)	20 (27.4)	32 (25.0)	15 (12.1)	0.011
Use antidiabetic agents					
Insulin, n (%)^b^	132 (40.6)	33 (45.2)	53 (41.4)	46 (37.1)	0.520
Types of insulin^b^					0.290
Bolus/basal insulin, n (%)	78 (24.0)	15 (20.5)	34 (26.6)	29 (23.4)	
Basal insulin, n (%)	54 (16.6)	18 (24.7)	19 (14.8)	17 (13.7)	
Metformin, n (%)^b^	156 (48.0)	38 (52.1)	77 (60.2)	41 (33.1)	< 0.001
Sulfonylureas, n (%)^b^	39 (12.0)	10 (13.7)	17 (13.3)	12 (9.7)	0.597
Other antidiabetic drugs, n (%)^b^	126 (38.8)	29 (39.7)	56 (43.8)	41 (33.1)	0.216
Use antihypertensive agents					
RAAS inhibitors, n (%)^b^	56 (17.2)	16 (21.9)	28 (21.9)	12 (9.7)	0.018
CCBs, n (%)^b^	85 (26.2)	23 (31.5)	39 (30.5)	23 (18.5)	0.049
β-Blockers, n (%)^b^	24 (7.4)	6 (8.2)	12 (9.4)	6 (4.8)	0.370
Use lipid-lowering agents					
Statins, n (%)^b^	85 (26.2)	26 (35.6)	45 (35.2)	14 (11.3)	< 0.001
Fibrates, n (%)^b^	4 (1.2)	3 (4.1)	1 (0.8)	0 (0)	0.034

Data are number of subjects (percentage) or medians (interquartile ranges).

*SBP* systolic blood pressure, *DBP* diastolic blood pressure, *BMI* body mass index, *TC* total cholesterol, *TG* triglyceride, *ALT* alanine aminotransferase, *AST* aspartate aminotransferase, *GGT* γ-glutamyltransferase, *BUN* blood urea nitrogen, *SCr* serum creatinine, *eGFR* estimated glomerular filtration rate, *CGM* continuous glucose monitoring, *SD* standard deviation, *MAGE* mean amplitude of glucose, *CV* coefficient of variation, *TIR* time in range, *25OHD* 25-hydroxyvitamin D, *ASCVD* atherosclerotic cardiovascular disease, *RAAS inhibitors* inhibitors of the renin–angiotensin–aldosterone system, *CCBs* calcium channel blockers.

^a^Calculated using by analysis of variance.

^b^Calculated using the χ^2^ test.

^c^Calculated using the Wilcoxon signed rank test.

### Glucose metabolism metrics characteristics by serum 25OHD levels

**Figure 2:**
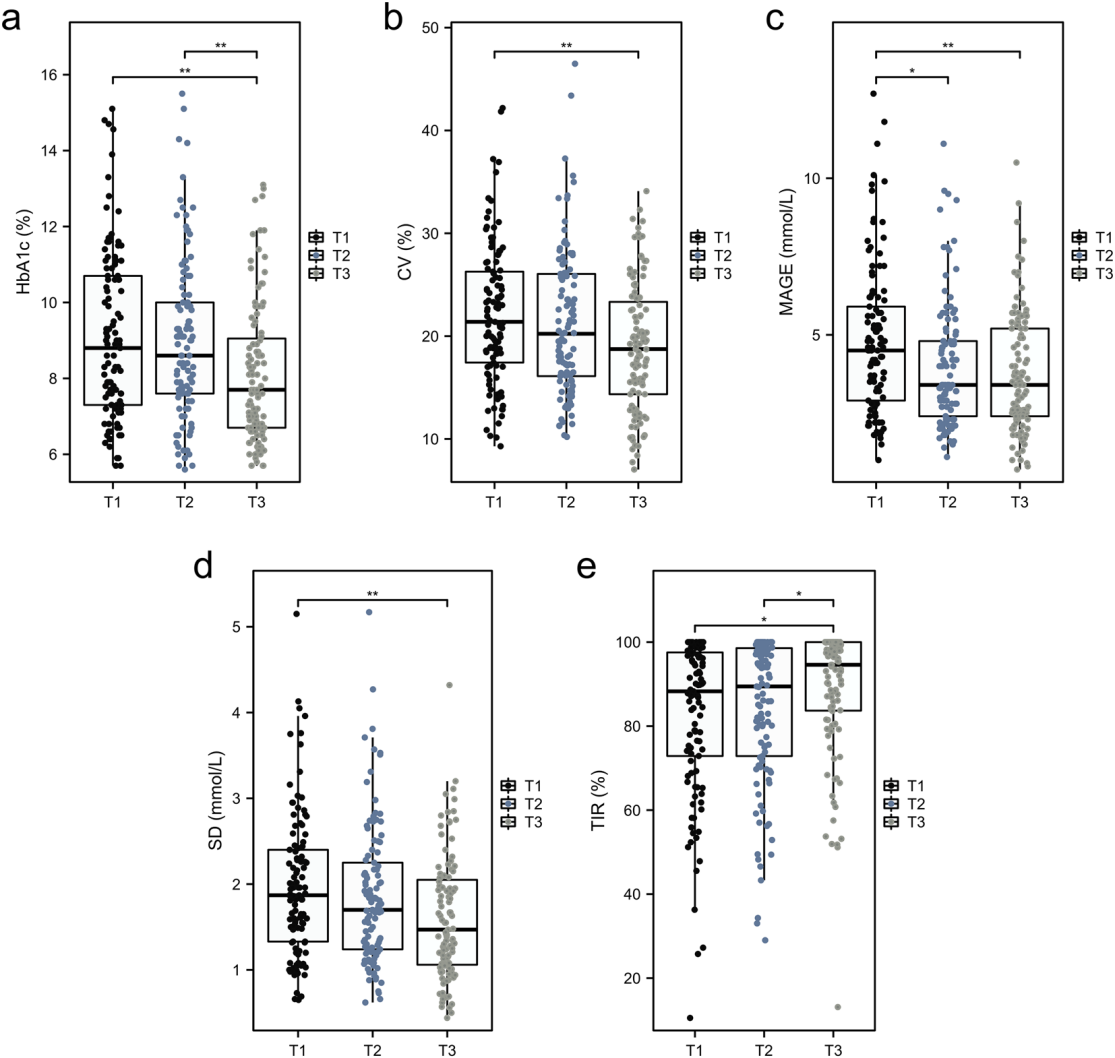
Comparisons of HbA1c and metrics of glucose variability among 25OHD tertiles, (**A**) HbA1c (%), (**B**) MAGE (mmol/L), (**C**) CV (%), (**D**) SD (mmol/L), (**E**) TIR (%). T1 (≤ 10.42 ng/mL), T2 (10.43–13.73 ng/mL), T3 (≥ 13.74 ng/mL). *P < 0.05; **P < 0.01.

The average mean 25OHD level of all the participants was 13.18 ng/mL, and the incidence of vitamin D deficiency (< 20 ng/mL) was 90.46%. Across 25OHD tertile (T1 < 10.42 ng/mL, T2[10.42–13.73 ng/mL] and T3 ≥ 13.74 ng/mL), HbA1c was statistically lower in the T3 group than in the other two groups (vs. T1, *P* = 0.001; vs. T2, *P* = 0.003). The SD in the T3 group was significantly lower than that T2 group (*P* = 0.025) and T1 group (*P* = 0.001). CV decreased significantly in the T3 group compared with the T1 and T2 groups (vs. T1, *P* = 0.001; vs. T2, *P* = 0.014). TIR was higher in the T3 group than in the other two groups (vs. T1, *P* = 0.011; vs. T2, *P* = 0.045). MAGE in the T1 group was significantly higher than that in the other two groups (vs. T2, *P* = 0.005; vs. T3, *P* = 0.005) (Fig. 2).

### Correlation of 25OHD with HbA1c and CGM parameters

**Figure 3:**
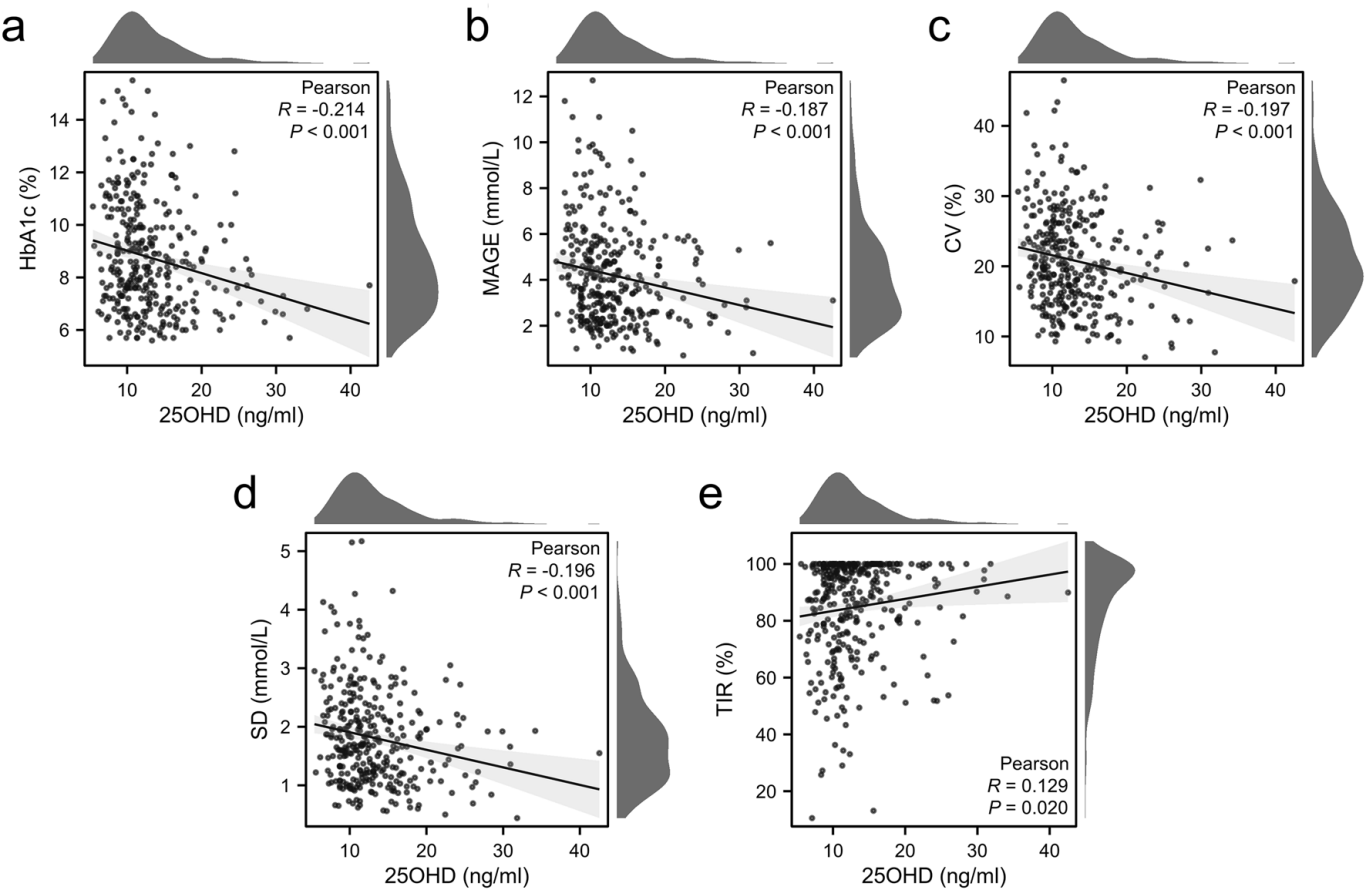
Correlations between 25OHD and HbA1c and glycemic variability metrics levels, (**A**) HbA1c (%), (**B**) MAGE (mmol/L), (**C**) CV (%), (**D**) SD (mmol/L), (**E**) TIR (%). *P < 0.05; **P < 0.01.

Figure 3 describes the linear correlation between glucose-related indices and 25OHD. There was a significantly negative association of HbA1c (r = − 0.214, *P* < 0.001), SD (r = − 0.196, *P* < 0.001), CV (r = − 0.197, *P* < 0.001) and MAGE (r = − 0.187, *P* < 0.001) with 25OHD. TIR rose statistically as 25OHD decreased (r = 0.129, *P* = 0.020).

### Association of 25OHD with HbA1c and CGM parameters

**Figure 4:**
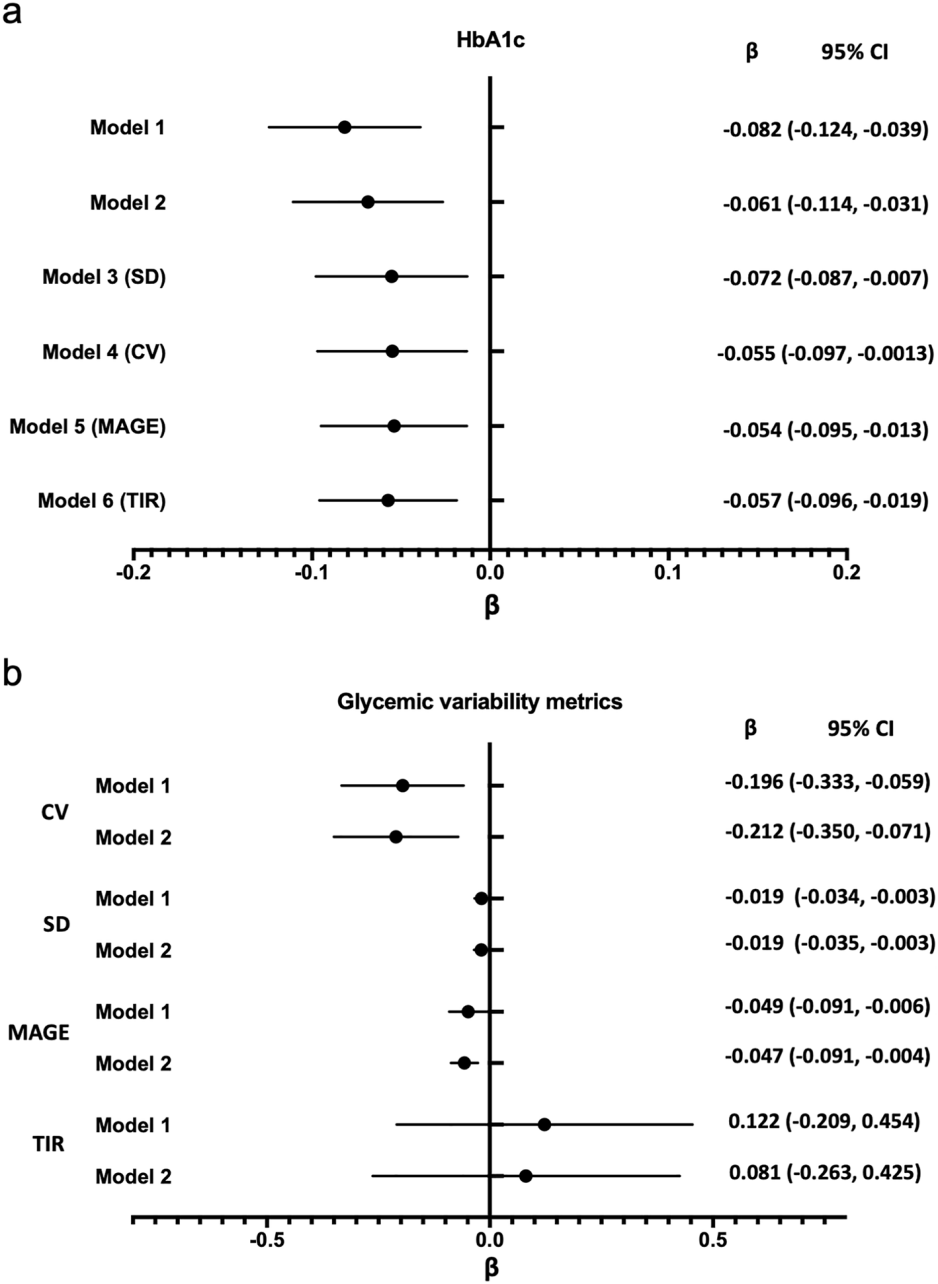
Multivariable-adjusted associations of measures of 25OHD with HbA1c and glycemic variability metrics. (**A**) Associations of measures of 25OHD with HbA1c. Model 1 was adjusted for sex, age, BMI, DM duration, SBP, and DBP; Model 2 was adjusted for model 1 + TC, TG, eGFR, cardiovascular and cerebrovascular disease, antidiabetic agents, antihypertension agents and lipid-lowering agents; Model 3 was adjusted for model 2 + SD; Model 4 was adjusted for model 2 + CV; Model 5 was adjusted for model 2 + MAGE; and Model 6 was adjusted for model 2 + TIR. (**B**) Associations of measures of 25OHD with glycemic variability metrics. Model 1 was adjusted for sex, age, BMI, DM duration, SBP, DBP, and HbA1c; Model 2 was adjusted for model 1 + TC, TGs, eGFR, cardiovascular and cerebrovascular disease, antidiabetic agents, antihypertension agents and lipid-lowering agents. Point estimates (unstandardized β [β]) and 95% CIs represent the difference (in SD) in HbA1c or glycemic variability metrics per SD increase in the measure of 25OHD.

As shown in Fig. 4a, multivariate linear regression showed that HbA1c was inversely associated with serum 25OHD after adjustment for age, sex, DM duration and blood pressure (Model 1: β = − 0.082 [− 0.124 to − 0.039], *P* < 0.001). The linear trend for HbA1c across serum 25OHD levels remained after further adjustment for potential confounders and mediators, including lipids, renal function, atherosclerotic cardiovascular disease history, medication history and parameters for GV (Model 2–6). In terms of these GV metrics (Fig. 4b), only CV had a significant (negative) correlation with 25OHD (Model 1: β = − 0.196 [− 0.333 to − 0.059], *P* = 0.005; Model 2: β = − 0.211 [− 0.350 to − 0.071], *P* < 0.01). SD and MAGE also revealed an inverse relationship with 25OHD after adjustment for covariates with statistical significance close to 0.05 (SD: β = − 0.019 [− 0.035 to − 0.003], *P* = 0.018; MAGE: β = − 0.047 [− 0.091 to − 0.004], *P* = 0.033). There were no significant associations between TIR and serum 25OHD.

These findings were further confirmed by multiple logistic regression models. All participants were categorized into two groups according to median of 25OHD levels at the cutoff point of 11.77 ng/mL and analyzed as categorical variables. As indicated in Table 2, an association between lower HbA1c level (< 7%) and higher 25OHD was discovered after adjustment for demographic and other metabolic confounders (OR = 0.380 [0.208–0.696], *P* = 0.002). The participants were classified into two equal segments according to CV, SD and MAGE with cut-off point of 19.87%, 1.69 mmol/L and 3.8 mmol/L, respectively, as for GV metrics, only CV had a significant correlation with 25OHD (OR = 0.516(0.310–0.859), *P* = 0.011).

**Table 2:** Association of 25OHD with HbA1c and CGM metrics(25OHD as categorical variable).

	25OHD (ng/mL)		
	< 11.77	≥ 11.78	*P*
HbA1c			
Model 1^a^	1.0 (ref)	0.454 (0.261–0.790)	0.005
Model 2^a^	1.0 (ref)	0.380 (0.208–0.696)	0.002
CV			
Model 1^a^	1.0 (ref)	0.514 (0.317–0.834)	0.007
Model 2^a^	1.0 (ref)	0.516 (0.310–0.859)	0.011
SD			
Model 1^a^	1.0 (ref)	0.755 (0.460–1.240)	0.267
Model 2^a^	1.0 (ref)	0.771 (0.453–1.311)	0.336
MAGE			
Model 1^a^	1.0 (ref)	0.718 (0.446–1.156)	0.173
Model 2^a^	1.0 (ref)	0.743 (0.449–1.228)	0.246

Model 1 was adjusted for age, gender, BMI, DM duration, SBP and HbA1c (HbA1c would be adjusted if it was not independent variable).

Model 2 was adjusted for model 1 + TC, TG, eGFR, ASCVD, antidiabetic agents, antihypertension agents and lipid-lowering agents.

^a^Calculated using multivariate binary logistic regression.

Studies have shown that HbA1c does not adequately represent glycemic control in patients with high glycemic CV, in view of this, the association between 25OHD and HbA1c was analyzed at different tertiles of glucose CV (Table 3). The negative relationship between 25OHD and HbA1c still existed in the lowest and middle tertile groups after adjustment for demographic and other metabolic confounders (*P* < 0.05). However, the associations between 25OHD and HbA1c were attenuated in the highest tertile (Model 1: β = − 0.033 [− 0.113, − 0.048], *P* = 0.422; Model 2: β = − 0.040[− 0.125, 0.044], *P* = 0.346).

**Table 3:** Association of 25OHD with HbA1c after CV stratification.

	Model	β coefficient	95% CI	*P* value
CV				
≤ 17.25%	Model 1^a^	− 0.080	(− 0.147, − 0.013)	0.020
	Model 2^a^	− 0.070	(− 0.137, 0.002)	0.042
17.26–23.31%	Model 1^a^	− 0.092	(− 0.168, − 0.017)	0.018
	Model 2^a^	− 0.083	(− 0.160, 0.005)	0.037
≥ 23.32%	Model 1^a^	− 0.033	(− 0.113, − 0.048)	0.422
	Model 2^a^	− 0.040	(− 0.125, 0.044)	0.346

Model 1 was adjusted for age, gender, BMI, DM duration, SBP, DBP.

Model 2 was adjusted for model 1 + TC, TG, eGFR, atherosclerotic cardiovascular disease, antidiabetic agents, antihypertension agents and lipid-lowering agents.

^a^Calculated using multivariate linear regression analysis.

## Discussion

In the cross-sectional study of 325 patients with T2DM, we observed that higher glycemic variability derived from CGM and HbA1c were both associated with lower serum 25OHD levels. For glycemic variability measures, the association of glucose CV with 25OHD was independent of a broad array of potential confounders. Moreover, a null association of HbA1c and vitamin D was found in patients with a higher glucose CV, which suggests that glucose CV should be taken into consideration when assessing the correlation between glycemic control and vitamin D levels.

Our study proved HbA1c was inversely correlated with 25OHD level, moreover glycemic variability was also inversely correlated with 25OHD. Glycemic variability is now an important measure for glycemic management in T2DM^17,18^. Evidence has confirmed the association of higher glycemic variability had an increased risk of macrovascular and microvascular complications, likely due to oxidative stress and subsequent endothelial dysfunction induced by glucose swings^19,20^. In addition, recent studies have suggested that GV may increase the risk of cognitive dysfunction and dementia^21^. Despite this, few studies have assessed the correlation between vitamin D and glycemic variability in patients with T2DM. A growing number of epidemiological and clinical studies have shown that vitamin D has a wide influence on extraskeletal activities^22,23^. Meanwhile vitamin D deficiency (< 20 ng/mL) is increasing rapidly due to sun-protective habits, excessive indoor activities, and overweight/obesity^24^. The prevalence of vitamin D deficiency in the general population ranges from 20 to 100%^25,26^, whereas the prevalence in T2DM patients is more than 2 times that in the general population^27^. In this study, among the metrics of glycemic variability, CV, SD and MAGE were decreased with the increasing 25OHD levels, and the trend was the opposite for the TIR. In addition, the correlation analysis validated that 25OHD was negatively associated with HbA1c and metrics of GV in T2DM. Among the metrics of GV, only CV remained significant even after adjusting for HbA1c and other factors, and the findings were similar in multivariable logistic regression analyses. This suggested that the association between short-term fluctuations in glycemia and vitamin D deficiency warrants further investigation. In the future, cohort studies are needed to confirm whether vitamin D can improve glycemic variability in patients with T2DM.

Vitamin D is essential to maintain blood glucose levels. Clinical investigation and basic research provides a rational basis for vitamin D to maintain the stability of blood glucose. Vitamin D3 (VitD3) is the active form of vitamin D in plasma and it is thought to contribute to normal insulin release in β cells. The active form of vitD3 plays an important regulatory role in different cellular ionic homeostasis, while the process of insulin secretion is regulated by the ionic calcium-dependent mechanism. Moreover, a specific response element for vitD3 was detected in the insulin gene promoter, and vitamin D receptor and vitamin D-binding proteins as well as enzyme 1-α hydroxylase (responsible for the activation of vitD3) was detected in pancreatic tissues, suggesting the important role of vitamin D in the improvement of β-cell function and peripheral insulin sensitivity^28–30^. Additionally, other pathways may be involved in the antidiabetic effects of vitD3; for example, vitD3 has modulatory effects on the insulin signaling pathway and may improve the efficiency of pancreatic islets and prevent insulin signal transduction damage with its antioxidative and anti-inflammatory activities^31–34^. Studies indicate a negative correlation between glycemic variability and the secretion and action of insulin, and reduced responsiveness of beta cells to glucose stimulation as well as reduced responsiveness of peripheral tissues to insulin may lead to increased glycemic variability^35^. The effect of vitamin D on glycemic variability may occur through the influence of insulin secretion and insulin target organs such as skeletal muscle.

The glucose CV is the primary metric of short-term glycemic variability, it (but not SD) was found to be highly associated with hypoglycemia in type 1 diabetes^36,37^. A recent study showed that higher glucose CV was associated with increased in-hospital mortality and length of stay in patients with COVID-19^38^. There is increasing recognition that HbA1c cannot adequately represent glycemic control, especially in patients with higher glucose CV^39^. Interestingly, HbA1c levels and all-cause mortality rates appears to be weakened in individuals with higher glucose CV, suggesting that glucose CV should systematically be taken into consideration when exploring this^40,41^. Hence, we explored the association between vitamin D and HbA1c in T2DM patients with different CV states separately. The significant correlation between vitamin D and HbA1c disappeared in patients with a high degree of glucose CV (*P* > 0.05). It is speculated that higher glucose CV may attenuate this correlation between 25OHD and HbA1c. Glucose CV is a combination of hypoglycemic and hyperglycemic fluctuations to describe glucose excursions relative to and independent of mean glycemia^36^. The CV has a weaker correlation with average blood glucose and HbA1c than other GV indicators, such as SD^42^. In addition, higher CV has been associated with hypoglycemia in adults with T2DM^43^. These factors may act against such a correlation across higher glucose CV. Given the inconsistencies in the results of vitamin D on glycemic improvement in previous studies^44^, stratified analysis by glucose CV subgroup may be worth exploring further.

The current study has several limitations. First, the sample size of this study is not large for a retrospective study, which means that longitudinal studies with larger sample sizes are needed in the future to obtain more convincing and accurate results. There still some confounding factors about vitamin D we did not investigated, such as sun exposure, physical exercise, dietary intake of certain foods (e.g., fortified dairy products or oily fish), calcium/vitamin D intake and insulin resistance. Third, as this was an observational study, we were unable to definitively establish a cause‒effect relationship of serum 25OHD and HbA1c or glycemic variability. Fourth, measures of glycemic variability derived from 3-day CGM data may not be sufficient to assess its representativeness for each patient. Although it has also been shown that within-day variability can be reliably assessed with 2- or 3-day CGM^45^, whether the interday variability can be accurately assessed also still requires further study. Finally, it should be noted that the present study included hospitalized patients who were at risk of acute stress, and circulating levels of 25(OH)D may not accurately reflect vitamin D status.

## Method

### Study population and design

This cross-sectional study consecutively recruited 325 adults with T2DM from hospitalized patients at the Department of Endocrinology and Metabolism of the Second Affiliated Hospital (Tangdu Hospital) of Air Force Medical University of China from January 2017 to December 2020. All included subjects were diagnosed with T2DM according to World Health Organization criteria^46^. Those subjects were equipped with CGM monitoring and were on stable hypoglycemic agents for the previous 3 months as well as laboratory tests while hospitalized. Other key exclusion criteria included other diabetes forms (e.g., type 1 diabetes, gestational diabetes mellitus), diabetic ketoacidosis, diabetic hyperglycemic hyperosmolar state, acute cardiovascular or cerebrovascular disease (myocardial infarction or stroke), and severe hepatic or renal dysfunction.

The study was approved by the Institutional Review Board of the Medical Ethics Committee of the Air Force Medical University of China in accordance with the tenets of the Declaration of Helsinki. All participants provided written informed consent prior to entering the study.

### CGM parameter measurements

A retrospective CGM system (Medtronic, Northridge, California, USA) was conducted for participants for 3 days. SMBG (capillary blood) levels were measured at least 4 times daily to calibrate the CGM. During CGM monitoring period, candidates were instructed to follow their routine diet and exercise habits but preferably to avoid vigorous exercise. After 3 days of monitoring, glucose fluctuations were assessed by GV metrics and TIR.

GV metrics were expressed as the standard deviation (SD) of sensor glucose values, the mean amplitude of glycemic excursions (MAGE) and the glucose coefficient of variation (CV). MAGE was defined as the average of absolute values of differences between adjacent peaks and nadirs for all differences > 1 SD. CV was calculated by the following formula: CV% = SD/mean blood glucose (MBG). TIR was computed by calculating the percentage of CGM glucose readings in the target range of 3.9–10.0 mmol/L.

### Anthropometric and biochemical evaluation

The height and weight of each participant were measured to calculate the BMI [= (kg/m^2^)]. Average blood pressure (BP) was calculated from three measurements more than 10 min at 3-min intervals with an automated electronic device (OMRON Model HEM-752 FUZZY, Omron Company, Dalian, China). Diabetes duration and use of antidiabetic, antihypertensive and lipid-lowering agents were assessed in the interviews. Blood samples were tested after 10 h fasting time to evaluate biochemical parameters. Blood lipids and indices of liver and renal function, including total triglyceride (TG) and total cholesterol (TC), albumin, globulin, alanine aminotransferase (ALT), aspartate aminotransferase (AST), alkaline phosphatase (ALP), γ-glutamyltransferase (GGT), blood urea nitrogen (BUN), serum creatinine (SCr), and estimated and serum uric acid (SUA), were measured by using an autoanalyser (ADVIA-1650 Chemistry System, Bayer Corporation, Germany). The estimated glomerular filtration rate (eGFR) was calculated according to the Modification of Diet in Renal Disease (MDRD)^47^. High-performance liquid chromatography for HbA1c was performed by using the VARIANT II Hemoglobin Testing System (Bio-Rad Laboratories, CA). Serum 25OHD was measured by chemiluminescence (Roche cobas e602, Rotkreuz, Switzerland) to assess vitamin D status. Participants with 25-hydroxyvitamin D < 20 ng/mL were considered deficient^6^.

### Statistical analysis

All statistical analyses were carried out by using SPSS V.26.0 and different packages of the R language (http://www.R-project.org/). The Kolmogorov‒Smirnov test was performed to assess the normality of quantitative data, among which data with a normal distribution are shown as the mean ± standard deviation (SD), while data with a skewed distribution are expressed as medians with interquartile ranges (25th–75th percentile). Categorical data were expressed as frequencies and percentages. ANOVA was performed to compare continuous variables with a normal distribution, and the Wilcoxon signed rank test was conducted to compare those with a skewed distribution. Categorical variables were compared using the χ^2^ test. Pearson correlation analysis and multivariate linear regression analysis were conducted to determine the association of serum 25OHD and metrics for glucose fluctuations, among which parameters with significant differences were chosen and further assessed by multivariate logistic regression to explore the association with 25OHD. A two-tailed *P* value < 0.05 suggested statistical significance.

### Ethical approval

The study was conducted in accordance with the Declaration of Helsinki and approved by the Ethics Committee of Tangdu Hospital (No. K202207-05). All subjects provided consent for their data to be used in this study.

### Informed consent

Written informed consent was obtained from all participants.

## Conclusions

In summary, higher HbA1c level and short-term glycemic variability of CV were both associated with lower 25OHD levels. Moreover, higher glucose CV may attenuate the association between HbA1c and 25OHD, which means glycemic variability should be taken into consideration when assess the correlation between the HbA1c and 25OHD, especially in patients featured by higher glucose CV. Long-term follow-up is needed to determine the benefits of vitamin D intervention on the reduction of HbA1c and GV.

## Data Availability

The datasets used and/or analyzed during the current study are available from the corresponding author on reasonable request.
